# Recurrence in traumatic anterior shoulder dislocations increases the prevalence of Hill–Sachs and Bankart lesions: a systematic review and meta-analysis

**DOI:** 10.1007/s00167-021-06847-7

**Published:** 2022-01-06

**Authors:** Cain Rutgers, Lukas. P. E. Verweij, Simone Priester-Vink, Derek F. P. van Deurzen, Mario Maas, Michel P. J. van den Bekerom

**Affiliations:** 1 Vrije Universiteit Amsterdam, De Boelelaan 1105, 1081 HV Amsterdam, The Netherlands; 2 Shoulder and Elbow Unit, Joint Research, Department of Orthopaedic Surgery, OLVG, Amsterdam, The Netherlands; 3 Medical Library, Department of Research and Epidemiology, OLVG, Amsterdam, The Netherlands; 4 Division of Musculoskeletal Radiology, Department of Radiology and Nuclear Medicine, Amsterdam UMC, Location AMC, University of Amsterdam, Amsterdam, The Netherlands

**Keywords:** Epidemiology, Prevalence, Shoulder instability, Shoulder dislocation, Glenoid labrum, Labrum lesions, Hill–Sachs

## Abstract

**Purpose:**

The extent of shoulder instability and the indication for surgery may be determined by the prevalence or size of associated lesions. However, a varying prevalence is reported and the actual values are therefore unclear. In addition, it is unclear whether these lesions are present after the first dislocation and whether or not these lesions increase in size after recurrence. The aim of this systematic review was (1) to determine the prevalence of lesions associated with traumatic anterior shoulder dislocations, (2) to determine if the prevalence is higher following recurrent dislocations compared to first-time dislocations and (3) to determine if the prevalence is higher following complete dislocations compared to subluxations.

**Methods:**

PubMed, EMBASE, Cochrane and Web of Science were searched. Studies examining shoulders after traumatic anterior dislocations during arthroscopy or with MRI/MRA or CT published after 1999 were included. A total of 22 studies (1920 shoulders) were included.

**Results:**

The proportion of Hill–Sachs and Bankart lesions was higher in recurrent dislocations (85%; 66%) compared to first-time dislocations (71%; 59%) and this was statistically significant (*P* < 0.01; *P* = 0.05). No significant difference between recurrent and first-time dislocations was observed for SLAP lesions, rotator-cuff tears, bony Bankart lesions, HAGL lesions and ALPSA lesions. The proportion of Hill–Sachs lesions was significantly higher in complete dislocations (82%) compared to subluxations (54%; *P* < 0.01).

**Conclusion:**

Higher proportions of Hill–Sachs and Bankart were observed in recurrent dislocations compared to first-time dislocations. No difference was observed for bony Bankart, HAGL, SLAP, rotator-cuff tear and ALPSA. Especially when a Hill–Sachs or Bankart is present after first-time dislocation, early surgical stabilization may need to be considered as other lesions may not be expected after recurrence and to limit lesion growth. However, results should be interpreted with caution due to substantial heterogeneity and large variance.

**Level of evidence:**

IV.

**Supplementary Information:**

The online version contains supplementary material available at 10.1007/s00167-021-06847-7.

## Introduction

Anterior shoulder dislocations commonly occur following a fall or direct impact to the shoulder at home or during sports/recreation activities, reporting an incidence of 23.9 per 100,000 person-years and making up 45% of all dislocations [1–3]. Over 95% of dislocations occur in the anterior direction, with the other 5% occurring in the posterior or inferior direction [4].

Traumatic anterior shoulder dislocations are often accompanied by lesions of the soft-tissue and bony structures in and around the glenohumeral joint [5]. Soft-tissue lesions are typically diagnosed through imaging, such as MRI or MRA, bony lesions through CT or CTA and ligamentous lesions through CTA or MRA. Compared to the gold standard arthroscopy, the sensitivity of imaging techniques varies between 60 and 95% soft-tissue lesions while CTA shows sensitivity and specificity close to 100% for bony lesions. [6–10]

Evidence regarding the prevalence of lesions is lacking. Systematic reviews determining the prevalence are limited and epidemiological studies show a varying range in the observed prevalence of lesions [11–13]. Multiple variables may attribute to this disagreement, such as the experience of the assessor, machine settings [14], accuracy of diagnostic tools [15], selection bias and unclear differentiation between first-time or recurrent (≥ 2) dislocation or subluxation [16]. A lack in epidemiological knowledge is problematic because the optimal management can vary between lesions. Little glenoid bone loss and low risk of recurrence may indicate a Bankart repair, whereas high glenoid bone loss and high risk of recurrence may indicate bony reconstruction such as the Latarjet procedure [17, 18]. Delayed surgical intervention, when this is needed, may cause a higher recurrence rate, degenerative changes and an increase in symptoms, especially in overhead and contact sports athletes [19–21]. Some lesions are more likely related to these outcomes than others, such as bony lesions, which are thought to be a risk factor for failure of conservative management and after soft-tissue stabilization [21, 22]. This makes the presence and size of bony lesions important for surgical decision-making. Epidemiological knowledge of associated lesions may allow professionals to anticipate on these lesions and assist in deciding on the optimal management [23–27].

Recent literature shows a paradigm shift from a more conservative approach to earlier surgical intervention to prevent recurrence and increase shoulder function [21, 28]. The presence and size of lesions may influence the extent of instability and determine the technique of surgical shoulder stabilization. Therefore, the aim of this systematic review was (1) to determine the prevalence of lesions associated with traumatic anterior shoulder dislocations, (2) to determine if the prevalence is higher following recurrent dislocations compared to first-time dislocations and (3) to determine if the prevalence is higher following complete dislocations compared to subluxations. It was hypothesized that lesions were more prevalent following recurrent dislocations compared to first-time dislocations and following complete dislocations compared to subluxations.

## Materials and methods

This systematic literature review was written according to the Preferred Reporting Items for Systematic Reviews and Meta-Analyses (PRISMA) statements, extended with a reporting standard to fit the needs for a systematic literature review [23]. This review has been registered to the Prospective Register of Systematic Reviews (PROSPERO registration ID: CRD42021233391, date of submission: 26-01-2021) [29].

### Search strategy

Relevant studies were identified by searching PubMed, Embase/Ovid, Cochrane Database of Systematic Reviews/Wiley, Cochrane Central Register of Controlled Trials/Wiley, and Web of Science/Clarivate Analytics by C.R. and S.P.V. The search was started and completed on December 17, 2020. The following terms, including synonyms and closely related words, were used as index terms or free-text words: ‘Shoulder dislocation’, ‘Arthroscopy’, ‘MRI’, ‘CT’ and terms for all included lesions. A filter was applied to find articles published from 2000 until at least the end of 2020. The complete search strategies can be found in Additional file 1.

### Study selection and quality assessment

Studies were independently assessed for eligibility by 2 authors (C.R. and L.P.E.V.), who individually screened the titles and abstracts using Rayyan (Hamad Bin Khalifa University, Doha, Qatar) [30]. Eligible studies were included in the full-text screening, which was also performed individually by 2 authors (C.R. and L.P.E.V.). Studies that met the inclusion criteria were included for analysis. Disagreement between authors was resolved by discussion and consensus.

Studies reporting the prevalence of associated lesions following traumatic anterior shoulder dislocations diagnosed lesions through arthroscopy, MRI, MRA, CT or CTA in a cohort of at least 10 patients were included. Studies written in English, Dutch or German were included. All cohorts had to be studied after the year 2000 due to the rapid progression of accuracy of diagnostic tools [31]. When case cohorts of two separate articles were overlapping, the article with the most different types of lesions was included. When this was the same between two articles, the article with the largest sample size was included. Cohort studies that followed patients after an intervention were only included when the prevalence of lesions for the entire population at the start of the follow-up period was reported.

Reviews, case reports, meta-analyses, biomechanical studies and cadaveric studies were excluded. Studies measuring the accuracy of diagnostic tools were excluded to prevent selection bias.

### Risk of bias

The risk of bias was independently assessed by 2 authors (C.R. and L.P.E.V.) using the Joanna Briggs Institute (JBI) standard for critical appraisal. This tool was designed for systematic reviews reporting prevalence data [32]. Disagreement between authors was resolved by discussion and consensus.

### Identification of lesions

Arthroscopy was considered the gold standard for all lesions. When lesions were not identified by arthroscopy, MRI or MRA was considered the highest ranking modality for soft-tissue lesions and CT or CTA was considered the highest ranking modality for bony lesions.

### Data extraction

Baseline characteristics included sample size, male/female ratio, mean age with range and standard deviation (SD), indication for inclusion (e.g., arthroscopic surgery), highest ranking modality and population characteristics (e.g., athletes or military personnel). The primary outcome was the prevalence of lesions associated with traumatic anterior shoulder dislocations. The included lesions were defined in Table 1 in cooperation with an experienced musculoskeletal radiologist (M.M.) and orthopedic surgeon that specializes in shoulder and elbow pathology (M.P.J.B.). The authors of the included articles may not have used the same definitions. Secondary outcomes include the number of dislocations, whether patients experienced a complete dislocation or subluxation and whether patients experienced a recurrent dislocation (≥ 2) or first-time dislocation. A dislocation was defined as any type of anterior dislocation, including subluxations and complete dislocations. A complete dislocation was defined as an anterior dislocation requiring manual reduction. All data were extracted and transferred by 1 Author (C.R.) to Excel (Microsoft Corporation. Microsoft Excel [Internet]. 2012. Available from: https://office.microsoft.com/excel). To evaluate the reliability and completeness of the data extracted by C.R., the data from a random sample of 10 articles were independently extracted and transferred by Author 2 (L.P.E.V.) to an independent Excel database. The two databases were compared by both authors. Both databases corresponded with each other.

**Table 1:** Included lesions and definitions

Lesions	Definition
Bony lesions	
Hill–Sachs	Impression fracture of the posterolateral humeral head
Glenoid lesion	Depressed or raised surface of the glenoid
Loose bodies	A loose osseous fragment inside the glenohumeral joint, originating from the glenoid rim or humeral head
Bony Bankart	A bony lesion or fracture involving the anterior labrum and glenoid rim
Soft-tissue lesions	
Bankart	A tear of the anterior labrum at 2–6 o’clock position
Posterior Bankart	A tear of the posterior labrum at 6–11 o’clock
Perthes	Anteroinferior labrum is partially detached and periosteum is stripped medially but still intact
Anterior Labral Periosteal Sleeve Avulsion (ALPSA)	The labroligamentous complex is displaced medially; however, the labrum and glenoid rim are still intact
Superior Labral tear from Anterior to Posterior (SLAP tear)	A tear of the superior labrum at 11–2 o’clock position
GlenoLabral Articular Disruption (GLAD)	The labroligamentous complex is partially teared and the cartilage is damaged
Capsular lesions	A lesion to the shoulder joint capsule including the following lesions: HAGL, GAGL, AIGHL, IGHL, PHAGL
Humeral Avulsion of the Glenohumeral Ligament (HAGL)	Avulsion fracture of the inferior glenohumeral ligament at the humeral insertion
Glenoid Avulsion of the Glenohumeral Ligament (GAGL)	Avulsion fracture of the inferior glenohumeral ligament at the glenoid insertion
Anterior Inferior GlenoHumeral Ligament avulsion(AIGHL)	Avulsion fracture of the anterior inferior glenohumeral ligament
Inferior GlenoHumeral Ligament avulsion (IGHL)	Avulsion fracture of the inferior glenohumeral ligament
Posterior Humeral Avulsion of the Glenohumeral Ligament (PHAGL)	Avulsion fracture of the posterior inferior glenohumeral ligament at the humeral insertion
Rotator-cuff tear	A tear of the m. Subscapularis, m. Infraspinatus, m. Supraspinatus or m. Teres minor
Chondral lesion	Chondral injury of the glenoid or humeral head
Long head of the biceps tear	(Partial) tear of the long head of the biceps

### Statistical analysis

Baseline characteristics and outcome data were presented using descriptive statistics reported as mean and SD. In case the SD was not reported, it was estimated using the sample size and range according to Walter et al.[33]. The data were pooled and a weighted mean was calculated. Proportions of lesions were analyzed for the amount of dislocations (1, ≥ 2) and type of dislocation (complete dislocation, subluxation). Other outcomes, such as modality, age, patient characteristics and indication, could not be analyzed because there were not enough data. Instead, these outcomes were described. When the prevalence of a lesion was not reported in an article, it did not take part in the analysis. Proportions were compared with chi-square tests. Confidence intervals (CI) were reported where possible. Odds-ratios (ORs) and 95% CI were calculated with Review Manager version 5.3 (the Nordic Cochrane Center, Copenhagen, Denmark). *P *values ≤ 0.05 were considered significant. Heterogeneity between studies was determined by the *I*^2^ value. *I*^2^ > 50% was considered to signify substantial heterogeneity [34].

## Results

### Screening and study characteristics

**Fig. 1:**
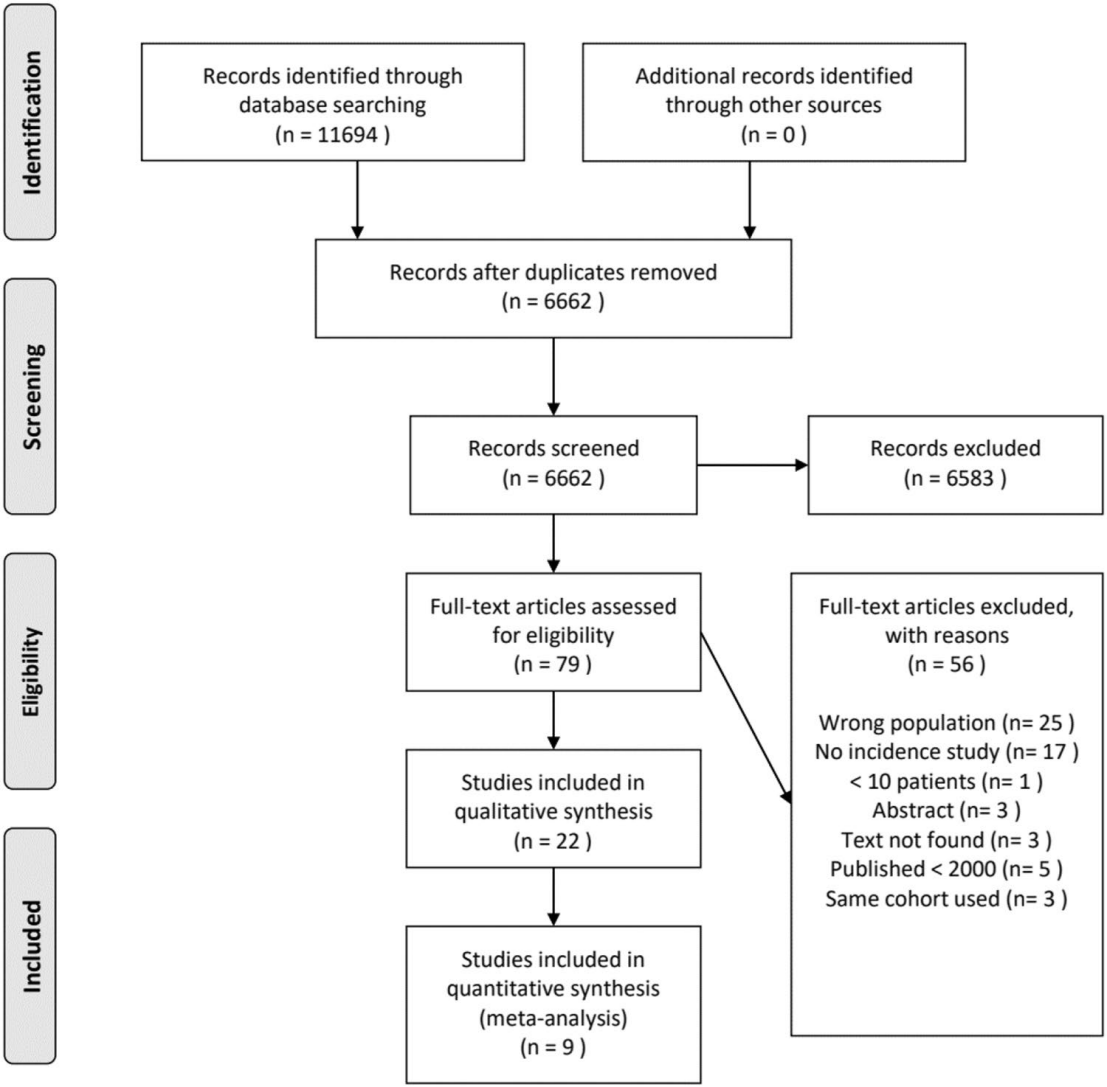
PRISMA Flow diagram[60]

After duplicates were removed, 6662 studies were screened by title and abstract. The full-text of 79 articles was read to determine if the article was eligible for inclusion. The search strategy resulted in 22 articles meeting the inclusion criteria. Reasons for exclusion during full-text screening are shown in Fig. 1.

Seven studies had a prospective design and 15 studies had a retrospective design (Additional file 2: Table 1). Arthroscopy was the modality of choice in 15 studies. The highest ranking modality was MRI or MRA in four studies and CT in two studies. One MRI study identified soft-tissue and bony lesions while the other three identified only soft-tissue lesion. Both CT studies identified only bony lesions. Athletes were studied in three articles and military personnel was studied in two articles. A population older than 35 was studied in two articles. The indication for inclusion was surgery in 13 articles, diagnostic imaging in six articles, a dislocation event in two articles and diagnostic arthroscopy in one article.

A total of 1920 shoulders were studied for lesions associated with traumatic anterior shoulder dislocation. The pooled mean age was 26.2 ± 8.9 years. There were 581 complete dislocations from 6 studies, 101 subluxations from 3 studies, 1152 recurrent dislocations from 15 studies and 392 first-time dislocations from 10 studies. For every dislocation, 1.8 lesions were identified. Lesions were determined using arthroscopy in 72% of shoulders and imaging in 28% of shoulders.

### Quality control

Four articles had one question of the JBI critical appraisal answered with ‘No’. All other questions were answered with ‘Yes’. Question 1, regarding the appropriateness of the studied sample was answered with ‘No’ for Takase et al. Their objective was to investigate lesions in all shoulders; however, they only included patients undergoing Bankart repair. Question 4, regarding how detailed the study setting was described was answered with ‘No’ for Ozaki et al. Their methods describe the presence of Hill–Sachs as inclusion criteria, however from the results can be concluded that a general population was studied, including without Hill–Sachs. Question 7, regarding the reliability of measurements and standardized methods for all patients was answered with ‘No’ for Owens et al. and Kim et al. Both studies diagnosed part of their population by arthroscopy and part by imaging (Additional file 2: Table 2). Two authors (C.R. and L.P.E.V.) completed the JBI critical appraisal independently of each other. Minor differences were found which were resolved by discussion and consensus.

### Prevalence of bony lesions

In a pooled sample of 1,845 shoulders from 22 studies, a bony lesion was reported in 96%. A Hill–Sachs (69%) lesion was most prevalent and bony Bankart (13%) least prevalent (Table 2). The lesion prevalence found in each individual study was summarized in Additional file 2: Table 3.

**Table 2:** Prevalence of bony lesions

	Studies (*n*)	Shoulder (*n*)	Prevalence (%)	Range (%)
Hill–Sachs	20	1731	69	13–100
Bony glenoid	10	983	37	6–86
Loose body	7	566	15	9–44
Bony Bankart	8	889	13	0–43

### Prevalence of soft-tissue lesions

In a pooled sample of 1320 shoulders from 17 studies, a labral lesion was reported in 97% of shoulders. A Bankart (67%) lesion was most prevalent, followed by posterior Bankart (23%) and SLAP (23%) lesions (Table 3). A Perthes lesion was least prevalent (14%).

**Table 3:** Prevalence of labral lesions

	Studies (*n*)	Shoulder (*n*)	Prevalence (%)	Range (%)
Bankart	14	993	67	20–100
Posterior Bankart	3	204	23	3–42
Perthes	3	79	14	0–32
ALPSA	8	542	18	0–26
SLAP	15	1,245	23	0–64
GLAD	4	355	4	0–20

In a pooled sample of 1018 shoulders from 11 studies, a capsular lesion was reported in 16% (Table 4).

**Table 4:** Prevalence of capsular lesions

	Studies (*n*)	Shoulder (*n*)	Prevalence (%)	Range (%)
HAGL	10	988	3	1–21
AIGHL	1	30	90	N.a
IGHL	1	42	33	N.a
PHAGL	1	42	31	N.a
GAGL	1	25	40	N.a

In a pooled sample of 1634 shoulders from 19 studies, there were 1.3 soft-tissue lesions per shoulder. Excluding labral lesions, a rotator-cuff tear (17%) was most prevalent (Table 5). A long head of the biceps lesion was least prevalent. (8%).

**Table 5:** Prevalence of soft-tissue lesions

	Studies (*n*)	Shoulder (*n*)	Prevalence (%)	Range (%)
Rotator-cuff tear	13	1290	17	2–64
Chondral	3	325	9	4–28
Long head of the biceps	3	257	8	5–18

### Meta-analyses of lesion prevalence for first-time and recurrent dislocations

**Fig. 2:**
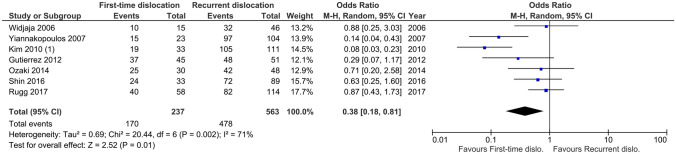
Meta-analysis of Hill–Sachs first-time vs recurrent proportions. This meta-analysis shows the odds ratio for studies (*n* = 7) that reported the prevalence of Hill–Sachs lesions in first-time dislocations compared to recurrent dislocations

A lower proportion of Hill–Sachs lesions was observed following first-time dislocation (71%, range 58–83%) compared to recurrent dislocation (85%, range 70–95%; *P* = 0.01, *I*^2^ = 71%; Fig. 2).

**Fig. 3:**
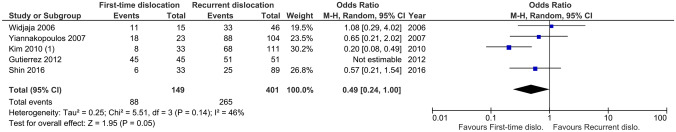
Meta-analysis of Bankart first-time vs recurrent dislocation proportions. This meta-analysis shows the odds ratio for studies (*n* = 5) that reported the prevalence of Bankart lesions in first-time dislocations compared to recurrent dislocations

A lower proportion of Bankart lesions was observed following first-time dislocation (59%, range 18–100%) compared to recurrent dislocation (66%, range 28–100%; *P* = 0.05, *I*^2^ = 46%; Fig. 3).

**Fig. 4:**
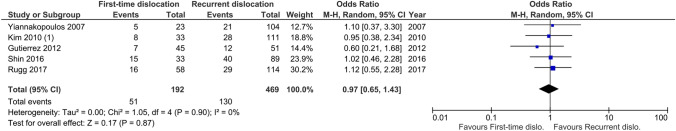
Meta-analysis of SLAP first-time vs recurrent dislocation proportions. This meta-analysis shows the odds ratio for studies (*n* = 5) that report the prevalence of SLAP lesions in first-time dislocations compared to recurrent dislocations

No significant difference was observed for SLAP lesions following first-time dislocation (27%, range 16–45%) compared to recurrent dislocation (28%, range 20–45%; *P* = n.s., *I*^2^ = 0%; Fig. 4).

**Fig. 5:**
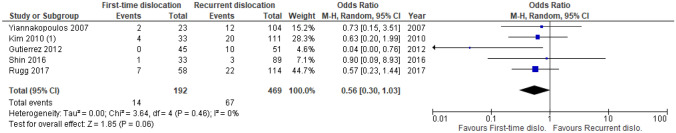
Meta-analysis of rotator-cuff tear first-time vs recurrent dislocation proportions. This meta-analysis shows the odds ratio for studies (*n* = 5) that report the prevalence of rotator-cuff tears in first-time dislocations compared to recurrent dislocations

No significant difference was observed for partial- or full thickness rotator-cuff tears following first-time dislocation (7%, range 0–12%) compared to recurrent dislocation (14%, range 3–20%; *P* = n.s., *I*^2^ = 0%; Fig. 5).

**Fig. 6:**
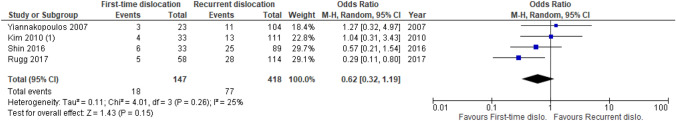
Meta-analysis of bony Bankart first-time vs recurrent dislocation proportions. This meta-analysis shows the odds ratio for studies (*n* = 4) that report the prevalence of bony Bankart lesions in first-time dislocations compared to recurrent dislocations

No significant difference was observed for bony Bankart lesions following first-time dislocation (12%, range 9–18%) compared to recurrent dislocation (17%, range 11–28%; *P* = n.s., *I*^2^ = 25%; Fig. 6).

**Fig. 7:**
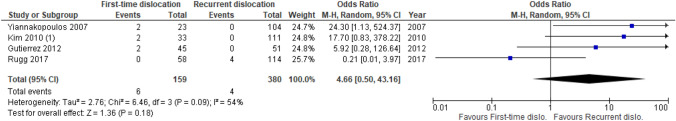
Meta-analysis of HAGL first-time vs recurrent dislocation proportions. This meta-analysis shows the odds ratio for studies (*n* = 4) that report the prevalence of HAGL lesions in first-time dislocations compared to recurrent dislocations

No significant difference was observed for HAGL lesions following first-time dislocation (4%, range 0–9%) compared to recurrent dislocation (1%, range 0–4%; *P* = n.s., *I*^2^ = 54%; Fig. 7).

**Fig. 8:**
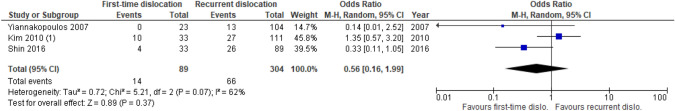
Proportions of ALPSA lesions for first-time dislocations compared to recurrent dislocations. This meta-analysis shows the odds ratio for studies (*n* = 3) that report the prevalence of ALPSA lesions in first-time dislocations compared to recurrent dislocations

No significant difference was observed for ALPSA lesions following first-time dislocation (16%, range 0–30%) compared to recurrent dislocation (22%, range 13–19%; *P* = n.s., *I*^2^ = 62%; Fig. 8).

### Meta-analysis of lesions prevalence for subluxations and complete dislocations

**Fig. 9:**
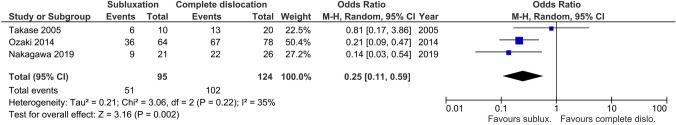
Meta-analysis of Hill–Sachs subluxation vs complete dislocation proportions. This meta-analysis shows the odds ratio for studies (*n* = 3) that report the prevalence of Hill–Sachs lesions in sub-dislocations compared to complete dislocations

A lower proportion of Hill–Sachs lesions was observed following subluxation (54%, range 43–60%) compared to complete dislocation (82%, range 65–86%; *P* < 0.01, *I*^2^ = 35%; Fig. 9).

## Discussion

The most important finding of the present study was that Hill–Sachs and Bankart lesions were found in higher proportions in recurrent dislocations compared to first-time dislocations. The prevalence of bony Bankart, HAGL, SLAP, rotator-cuff tear and ALPSA were similar when comparing recurrent- and first-time dislocations, suggesting these lesions may be more common after first-time dislocation than previously thought. However, results should be interpreted with caution due to the large range in prevalence; heterogeneity found in the Hill–Sachs, HAGL and ALPSA analyses; and limited amount of studies reporting on capsular lesions.

Current data may be valuable for health professionals in the outpatient clinic with regard to shared decision-making. The results may assist in estimating the risk of new lesions after recurrence, especially as literature on this topic is currently lacking. When Hill–Sachs and Bankart lesions are present, current results suggest that no other lesions may be expected after recurrence. Also, as the optimal management may differ between lesions, the epidemiological results may assist in anticipating which lesion is likely present and which type of surgical procedure may be indicated [35–39]. For optimal shared decision-making, the amount of dislocations and whether the dislocation was complete or not should be taken into consideration.

The initial size, location and the subsequent increase of a bony lesion after a recurrent dislocation may be an important factor in surgical decision-making. Hasegawa et al. found the number of dislocation events to be a predictor of the amount of bipolar bone loss [40]. A correlation between bony lesion size and risk of recurrence has been described by multiple authors and literature suggests early surgical intervention to decrease risk of recurrence and increase return-to-play [41, 42]. Current results may confirm this conclusion. Untreated traumatic rotator-cuff tears may cause more pain and dysfunction over time and together with capsulolabral damage may lead to increased instability [20]. Individual capsulolabral and HAGL lesions are also thought to play a role in glenohumeral instability [43–45]. The hypothesis that lesions increase in size after recurrence rather than in prevalence is supported by current results and literature. Literature suggests that instability increases over time in untreated shoulders even though current results suggest that most lesions may not increase in prevalence after recurrence. Health professionals may therefore need to consider early surgical stabilization after first-time dislocation to limit the increase in size of these lesions.

A varying prevalence was found between studies for most lesions. Challenges in classifying, measuring and defining lesions may attribute to this. The glenoid track method is used to determine the extent of glenoid bone loss but lacks a standardized method to produce the image, causing challenges with regard to reproducibility and the reliability [46]. It may be difficult to identify lesions of the labrum correctly, such as Bankart, as literature shows discrepancies in defining lesions according to the clockface-method [47, 48]. The Snyder classification is used to evaluate SLAP lesions but shows high inter-observer and intra-observer variability, even between experience specialists [49]. Lastly, the Outerbridge classification is used for the classification of chondral lesions but studied only limited times in small populations [50]. Extensions or revision of current classification systems may assist in better identification and evaluation of lesions, as was done by Maffet et al. for the SLAP lesion [51]. The varying prevalence may also be explained by demographical differences. This includes sports that are more popular in certain countries but also the glenoid morphology, which may differ between populations [52].

The term subluxation presents difficulties in interpretation as a clear method of identifying subluxations in practice is currently lacking. One can even question whether a Hill–Sachs lesion can be caused by subluxation as the (postero-superior) humeral head may not impact against the glenoid. When Hill–Sachs is present following subluxation the displacement may have been such that one should speak of a complete dislocation, perhaps with spontaneous reduction. Current literature suggests that false identification of Hill–Sachs lesions may be a result of normal ossification of the humeral head in developing shoulders, possibly explaining Hill–Sachs lesions following subluxation [53]. It is interesting that some studies identified posterior Bankart lesions in patients suffering from anterior dislocation. A systematic review by Ernat et al. discussed 270° and 360° arthroscopic repair in a group of patients who suffered from an isolated anterior instability, suggesting that posterior lesions may occur after anterior dislocation [54].

The large range in prevalence found in this review may also be partly explained by the varying accuracy of diagnostic tools between studies. The sensitivity and specificity of MRA compared to the gold standard arthroscopy for Perthes was found to be 67% and 100%, respectively, by Kehdr et al. and sensitivity was found to be 100% by Elkharbotly et al. [6, 8] Perthes and GLAD lesions may be difficult to diagnose because the labrum can remain in its natural position even though the labrum is torn [47, 55, 56]. Inexperienced assessors are thought to make less accurate and less reliable diagnoses, an effect that is enhanced for difficult to diagnose lesions, such as Perthes and GLAD [57, 58]. Accuracy may be improved by taking notice of subtle MR intensity changes or placing the arm in the ABER position, although the latter is debated due to increased scan time and discomfort [56, 59].

The results of our study should be interpreted with caution in light of the following limitations. First, some lesions were reported in only a small amount of studies, increasing the risk of selection bias. The meta-analysis of the bony Bankart, HAGL and ALPSA only included four or less studies and the prevalence of the AIGHL, IGHL, PHAGL and GAGL were determined from one study. In case of these lesions, the data may not be sufficient to draw firm conclusions. Second, there is a risk of selection bias because most studies included patients undergoing surgical intervention. In a patient group not undergoing surgical intervention, there may be a lower frequency of lesions. Third, demographical differences may affect the prevalence of lesions due to high-risk sports being popular in certain countries or varying glenoid morphology between populations [52]. Fourth, not all lesions may have been identified in retrospective arthroscopy studies because the procedure may have had a different goal, causing the surgeon to pay less attention to uncommon lesions. These limitations may be reflected by the varying prevalence found between studies and substantial heterogeneity.

Future research should focus on multiple aspects. First, determining the role of lesion size and lesion growth in evaluating risk of recurrence, return-to-play and surgical decision-making. This includes soft-tissue lesions and the role of posterior lesions following anterior dislocation. Literature suggests there may be a correlation but high level evidence is currently lacking. Second, creating standardized methods to diagnose and classify radiological and symptomatic lesions and to define the term subluxation. This may lower the observed variance. Third, determine the sensitivity and specificity of diagnostic tools in large and representative populations as this is currently debated in the literature. Fourth, for both soft-tissue lesions and bony lesions, the aim should be to increase the accuracy of the diagnosis for MRI. It is especially important to improve bone MRI so the patient is not exposed to the radiation from CT. Fifth, study the prevalence of shoulder lesions following anterior dislocation in larger and more populations to increase the value of future systematic reviews like this one. Lastly, demographical variations of glenoid morphology between populations should be studied, also in currently less represented populations.

## Conclusion

Higher proportions of Hill–Sachs and Bankart were observed in recurrent dislocations compared to first-time dislocations. No difference was observed for bony Bankart, HAGL, SLAP, rotator-cuff tear and ALPSA. Especially when a Hill–Sachs or Bankart is present after first-time dislocation, early surgical stabilization may need to be considered as other lesions may not be expected after recurrence and to limit lesion growth. However, results should be interpreted with caution due to substantial heterogeneity and large variance.

## Supplementary Information

Below is the link to the electronic supplementary material.

Supplementary file1 Additional file 1: File type: .pdf. This file includes the search terms used for all databases. (PDF 79 kb)

Supplementary file2 Additional file 2: File type: PDF. Table 1: Demographics of studies; Table 2: JBI Critical Appraisal Checklist for Prevalence Studies; Table 3: Prevalence of lesions per study (PDF 196 kb)

## Abbreviations

PRISMA: Preferred Reporting Items for Systematic Reviews and Meta-Analyses
JBI: Joanna Briggs Institute
CI: Confidence interval
SD: Standard deviation
