# Supplementary material for: Recurrence in traumatic anterior shoulder dislocations increases the prevalence of Hill–Sachs and Bankart lesions: a systematic review and meta-analysis
Source: Knee Surg Sports Traumatol Arthrosc. 2022 Jan 6;30(6):2130–40. doi: 10.1007/s00167-021-06847-7 (PMC9165262; doi:10.1007/s00167-021-06847-7)
Supplement: Supplementary file 1 — Supplementary file1 Additional file 1: File type: .pdf. This file includes the search terms used for all databases. (PDF 79 kb) [file 167_2021_6847_MOESM1_ESM.pdf]

Supplementary 1 : Search terms

All databases were searched on December 17th 2020. In all databases a filter was applied to find articles from 2000 until at least the end of 2020.

PubMed

#1	**Search:** ("Shoulder Dislocation"[Mesh] OR (("Shoulder"[Mesh] OR "Shoulder Joint"[Mesh] OR shoulder*[tiab] OR glenohumeral*[tiab]) AND ("Joint Dislocations"[Mesh] OR dislocat*[tiab] OR luxat*[tiab] OR subluxat*[tiab] OR instabilit*[tiab]))) **Sort by:** Most Recent	12,957
#2	**Search:** ("Arthroscopy"[Mesh] OR arthroscop*[tiab] OR "Surgical Procedures, Operative"[Mesh] OR "surgery"[Subheading] OR endoscop*[tiab] OR operative*[tiab] OR surger*[tiab] OR surgical*[tiab] OR scope*[tiab] OR keyhole*[tiab] OR "Magnetic Resonance Imaging"[Mesh] OR Magnetic-Resonance-Imaging*[tiab] OR MR-Imaging*[tiab] OR MRI[tiab] OR MRIs[tiab] OR NMR-imaging*[tiab] OR Magnetic-Resonance-Arthrograph*[tiab] OR MR-Arthrograph*[tiab] OR MRA[tiab] OR "Tomography, X-Ray Computed"[Mesh] OR CT[tiab] OR CTA[tiab] OR (compute*[tiab] AND tomograph*[tiab])) **Sort by:** Most Recent	5,463,380
#3	**Search:** ("Shoulder Injuries"[Mesh] OR Hill Sachs[tiab] OR "Bankart Lesions"[Mesh] OR Bankart*[tiab] OR ALPSA[tiab] OR HAGL[tiab] OR SLAP[tiab] OR GLAD[tiab] OR ((chondral*[tiab] OR bicep*[tiab] OR "Glenoid Cavity"[Mesh] OR glenoid*[tiab] OR Capsular*[tiab] OR "Rotator Cuff"[Mesh] OR Rotator-cuff*[tiab] OR anterior-labral-periosteal-sleeve*[tiab] OR glenohumeral-ligament*[tiab] OR Glenolabral*[tiab] OR labral*[tiab] OR Tuberosit*[tiab]) AND (tear*[tiab] OR lesion*[tiab] OR avulsion*[tiab] OR fracture*[tiab] OR "Fractures, Bone"[Mesh]))) **Sort by:** Most Recent	31,212
#4	Search: **#1 AND #2 AND #3** Sort by: **Most Recent**	6,235
#5	Search: **#1 AND #2 AND #3** Filters: **from 2000 - 2021** Sort by: **Most Recent**	4,085

OVID Embase

1	exp shoulder dislocation/ or exp recurrent shoulder dislocation/	9563
2	exp shoulder/	83344
3	(shoulder* or glenohumeral*).ti,ab,kw.	102179
4	exp joint dislocation/	4072
5	(dislocat* or luxat* or subluxat* or instabilit*).ti,ab,kw.	230461
6	2 or 3	138351
7	4 or 5	231426
8	6 and 7	15047
9	1 or 8	17274
10	exp arthroscopy/	32575
11	exp surgery/	5424367
12	su.fs.	2207003
13	exp nuclear magnetic resonance imaging/ or exp NMR imaging/	992174
14	exp computer assisted tomography/	1102620
15	(arthroscop* or endoscop* or operative* or surger* or surgical* or scope* or keyhole* or Magnetic-Resonance-Imaging* or MR-Imaging* or MRI or MRIs or Magnetic-Resonance-Arthrograph* or MR-Arthrograph* or NMR-imaging* or MRA or CT or CTA or (compute* and tomograph*)).ti,ab,kw.	4288599
16	10 or 11 or 12 or 13 or 14 or 15	7942085
17	exp shoulder injury/	18604

18	exp Bankart lesion/	519
19	(Hill Sachs or "Bankart" or ALPSA or HAGL or SLAP or GLAD).ti,ab,kw.	4512
20	exp rotator cuff/	9051
21	glenoid cavity/	1991
22	(chondral* or bicep* or glenoid* or Capsular* or Rotator-cuff* or anterior-labral-periosteal-sleeve* or glenohumeral-ligament* or Glenolabral* or labral* or Tuberosit*).ti,ab,kw.	81304
23	20 or 21 or 22	83517
24	exp fracture/	337935
25	(tear* or lesion* or avulsion* or fracture*).ti,ab,kw.	1705730
26	24 or 25	1786728
27	23 and 26	24701
28	17 or 19 or 27	37210
29	9 and 16 and 28	5440
30	limit 29 to yr="2000 - 2021"	4478

Cochrane Database of Systematic Reviews & Cochrane Central Register of Controlled Trials

#1	MeSH descriptor: [Shoulder Dislocation] explode all trees	143
#2	MeSH descriptor: [Shoulder] explode all trees	543
#3	MeSH descriptor: [Shoulder Joint] explode all trees	755
#4	(shoulder* or glenohumeral*):ti,ab,kw	12005
#5	#2 or #3 or #4	12005
#6	MeSH descriptor: [Joint Dislocations] explode all trees	689
#7	(dislocat* or luxat* or subluxat* or instabilit*):ti,ab,kw	8278
#8	#6 or #7	8390
#9	#1 or (#5 and #8)	895
#10	MeSH descriptor: [Arthroscopy] explode all trees	1486
#11	MeSH descriptor: [Surgical Procedures, Operative] explode all trees	118676
#12	MeSH descriptor: [] explode all trees and with qualifier(s): [surgery - SU]	58209
#13	MeSH descriptor: [Magnetic Resonance Imaging] explode all trees	7784
#14	MeSH descriptor: [Tomography, X-Ray Computed] explode all trees	5027
#15	(arthroscop* or endoscop* or operative* or surger* or surgical* or scope* or keyhole* or Magnetic-Resonance-Imaging* or MR-Imaging* or MRI or MRIs or Magnetic-Resonance-Arthrograph* or MR-Arthrograph* or MRA or NMR-imaging* or CT or CTA or (compute* and tomograph*)).:ti,ab,kw	1566
#16	#10 or #11 or #12 or #13 or #14 or #15	140332
#17	MeSH descriptor: [Shoulder Injuries] explode all trees	1044
#18	MeSH descriptor: [Bankart Lesions] explode all trees	10
#19	(Hill Sachs or Bankart or ALPSA or HAGL or SLAP or GLAD):ti,ab,kw	233
#20	MeSH descriptor: [Glenoid Cavity] explode all trees	10
#21	MeSH descriptor: [Rotator Cuff] explode all trees	347
#22	(chondral* or bicep* or glenoid* or Capsular* or Rotator-cuff* or anterior-labral-periosteal-sleeve* or glenohumeral-ligament* or Glenolabral* or labral* or Tuberosit*):ti,ab,kw	5216
#23	#20 or #21 or #22	5216
#24	MeSH descriptor: [Fractures, Bone] explode all trees	6083
#25	(tear* or lesion* or avulsion* or fracture*):ti,ab,kw	73530
#26	#24 or #25	73545
#27	#17 or #18 or #19 or (#23 and #26)	2121
#28	#9 and #16 and #27 with Cochrane Library publication date Between Jan 2000 and Dec 2020	81

Web of Science, Core collection

TOPIC (filter 2000-2020):
((shoulder* OR glenohumeral*) AND (dislocat* OR luxat* OR subluxat* OR instabilit*)) **AND** (arthroscop* OR endoscop* OR operative* OR surger* OR surgical* OR scope* OR keyhole* Magnetic-Resonance-Imaging* OR MR-Imaging* OR MRI OR MRIs OR Magnetic-Resonance-Arthrograph* OR MR-Arthrograph* OR MRA OR NMR-imaging* OR CT OR CTA OR (compute* AND tomograph*)) **AND** (Hill Sachs OR Bankart OR ALPSA OR HAGL OR SLAP OR GLAD OR ((chondral* OR bicep* OR glenoid* OR Capsular* OR Rotator-cuff* OR anterior-labral-periosteal-sleeve* OR glenohumeral-ligament* OR Glenolabral* OR labral* OR Tuberosit*) AND (tear* OR lesion* OR avulsion* OR fracture*)))

**Database**	**Before deduplication**	**After deduplicatione**
PubMed	4085	4079
OVID Embase	4478	1870
Cochrane Database of Systematic Reviews	5	2
Cochrane Central Register of Controlled Trials	76	4
Web of Science	3050	707
**Total**	11694	6662
