# Supplementary material for: Recurrence in traumatic anterior shoulder dislocations increases the prevalence of Hill–Sachs and Bankart lesions: a systematic review and meta-analysis
Source: Knee Surg Sports Traumatol Arthrosc. 2022 Jan 6;30(6):2130–40. doi: 10.1007/s00167-021-06847-7 (PMC9165262; doi:10.1007/s00167-021-06847-7)
Supplement: Supplementary file 2 — Supplementary file2 Additional file 2: File type: PDF. Table 1: Demographics of studies; Table 2: JBI Critical Appraisal Checklist for Prevalence Studies; Table 3: Prevalence of lesions per study (PDF 196 kb) [file 167_2021_6847_MOESM2_ESM.pdf]

Article title: Most lesions associated with traumatic anterior shoulder dislocations do not increase in prevalence after recurrence: A systematic review and meta-analysis of 1920 shoulders.

Journal name: Knee Surgery, Sports Traumatology, Arthroscopy (KSSTA)

Author names:

Affiliations:

E-mail address corresponding author:

Legends:

Table 1: Demographics of studies: File type: .pdf. This table describes the demographics of all included studies (n=22), sorted by publishing date. R.= Retrospective, P.= Prospective, M/F = Male/Female, SD = Standard Deviation.

Table 2: JBI Critical Appraisal Checklist for Prevalence Studies. File type: .pdf. This table shows the JBI critical appraisal for each individual study. The appraisal consists of nine question that can be answered with 'Yes', 'No', 'Unsure', 'Not applicable' or 'Unsure'. After answering these questions, a decision can be made to include or exclude the study. Q = Question, Y = Yes, N = No.

Table 3: Prevalence of lesions per study File type: .pdf. This table shows the prevalence of lesions in percentages for each individual study and a pooled average.

Table 1: Demographics of studies

Author (Year)	*Country*	*Design*	*N of shoulders*	*M/F ratio*	*Mean age (years) ± SD*	*Modality*	*Indication*	*Population characteristics*
Takase (2005)	Japan	P	30	26/4	28 ± 5.6	Arthroscopy	Bankart procedure	General population
Widjaja (2006)	Australia	R	61	41/20	34.1 ± 13.0	MRI	Diagnostic MRI	General population
Yiannakopoulos (2007)	Greece	P	127	127/0	23.8 ± 2.3	Arthroscopy	Operative arthroscopic	Males
Arrigoni (2008)	USA	R	33	29/4	25 ± 8.9	Arthroscopy	Latarjet procedure	General population
Kim (1) (2010)	South-Korea	P	144	137/7	24.8 ± 10.4	Arthroscopy	Diagnostic arthroscopic + MRA	Military
Liavaag (2010)	Norway	P	42	33/9	26.7 ± 1.1	MRI	Diagnostic MRI	General population
Owens (2010)	USA	R	14			Arthroscopy	Surgery	Military
Kim (2) (2010)	South-Korea	R	40	37/3	23.8 ± 4.2	MRA+ Arthroscopy	Diagnostic MRA	General population
Carrazzone (2011)	Brazil	R	57		27.3 ± 6.2	Arthroscopy	Surgery	General population
Gutierrez (2012)	Chile	R	96		28.2	Arthroscopy	Arthroscopic surgery	Athletes
Zhu (2013)	China	R	14	12/2	36.2	Arthroscopy	Arthroscopic surgery	Department of Sports medicine
Kurokawa (2013)	Japan	R	100	72/28	28 ± 10.1	CT	Diagnostic CT	General population
Ozaki (2014)	Japan	R	142	122/20	22.8 ± 15.8	Arthroscopy	Arthroscopic Bankart repair	Athletes
Atef (2015)	Egypt	P	240	176/64	35.2 ± 7.0	MRI	Traumatic anterior Dislocation	General population
Ellis (2015)	USA	R	114	87/27	15.13	Arthroscopy	Recurrent dislocation	General population
Robinson (2015)	USA	R	28	22/5	55 ± 9.6	Arthroscopy	Surgery	Older than 35
Shin (2016)	South-Korea	R	122	113/9	25.9 ± 3.9	Arthroscopy	Arthroscopic stabilization	General population
Madhu (2017)	India	P	25	23/2	24.7 ± 7.8	MRA	Diagnostic MRA	General population
Rugg (2017)	USA	P	172	144/28	25.3 ± 8.5	Arthroscopy	Surgical stabilization	General population
Nakagawa (2017)	Japan	R	172	146/26	23.3 ± 10.1	Arthroscopy	Bankart repair	General population
Nakagawa (2019)	Japan	R	47	41/6	17.5 ± 3.4	CT	Diagnostic CT	Athletes
Eren (2020)	Turkey	R	75	41/34	51.6 ± 6.2	Arthroscopy	Arthroscopic instability surgery	Between 40-60 years old

Table 2: JBI Critical Appraisal Checklist for Prevalence Studies.

Author	Q1	Q2	Q3	Q4	Q5	Q6	Q7	Q8	Q9	Overall appraisal
Takase (2005)	**N**	Y	Y	Y	Y	Y	Y	Y	Y	Include
Widjaja (2006)	Y	Y	Y	Y	Y	Y	Y	Y	Y	Include
Yiannakopoulos (2007)	Y	Y	Y	Y	Y	Y	Y	Y	Y	Include
Arrigoni (2008)	Y	Y	Y	Y	Y	Y	Y	Y	Y	Include
Kim (1) (2010)	Y	Y	Y	Y	Y	Y	Y	Y	Y	Include
Liavaag (2010)	Y	Y	Y	Y	Y	Y	Y	Y	Y	Include
Owens (2010)	Y	Y	Y	Y	Y	Y	**N**	Y	Y	Include
Kim (2) (2010)	Y	Y	Y	Y	Y	Y	**N**	Y	Y	Include
Carrazzone (2011)	Y	Y	Y	Y	Y	Y	Y	Y	Y	Include
Gutierrez (2012)	Y	Y	Y	Y	Y	Y	Y	Y	Y	Include
Zhu (2013)	Y	Y	Y	Y	Y	Y	Y	Y	Y	Include
Kurokawa (2013)	Y	Y	Y	Y	Y	Y	Y	Y	Y	Include
Ozaki (2014)	Y	Y	Y	**N**	Y	Y	Y	Y	Y	Include
Atef (2015)	Y	Y	Y	Y	Y	Y	Y	Y	Y	Include
Ellis (2015)	Y	Y	Y	Y	Y	Y	Y	Y	Y	Include
Robinson (2015)	Y	Y	Y	Y	Y	Y	Y	Y	Y	Include
Shin (2016)	Y	Y	Y	Y	Y	Y	Y	Y	Y	Include
Madhu (2017)	Y	Y	Y	Y	Y	Y	Y	Y	Y	Include
Rugg (2017)	Y	Y	Y	Y	Y	Y	Y	Y	Y	Include
Nakagawa (2017)	Y	Y	Y	Y	Y	Y	Y	Y	Y	Include
Nakagawa (2019)	Y	Y	Y	Y	Y	Y	Y	Y	Y	Include
Eren (2020)	Y	Y	Y	Y	Y	Y	Y	Y	Y	Include

Table 3: Prevalence of lesions per study

Author (year)	*Shoulders (n)*	*Hill-Sachs(%)*	*Bankart (%)*	*Bony Bankart (%)*	*ALPSA (%)*	*Capsular lesion (%)*	*SLAP (%)*	*GLAD (%)*	*Rotator-cuff tear (%)*	*Long head of the biceps lesion (%)*	*Glenoid lesion (%)*	*Chondral lesion (%)*	*Perthes (%)*	*Loose body (%)*
Takase (2005)	30	80	100	43		90	7							
Widjaja (2006)	61	69	72											
Yiannakopoulos (2007)	127	88	84	11	10	2	21		11		13			17
Arrigoni (2008)	33	76					64		6		6			6
Kim (1) (2010)	144	86	53	12	26	5	25	1	17					13
Liavaag (2010)	42	93	81		0	86	17							
Owens (2010)	14	50	93											
Kim (2) (2010)	40	55	20	8	28	15	20		10				8	10
Carrazzone (2011)	57	72	100				39		2	14	32	28		
Gutierrez (2012)	96	89	100			2	20		10			4		
Zhu (2013)	14	100	71	14	29		43	0	7		79		0	
Kurokawa (2013)	100	94									86			
Ozaki (2014)	142	83				16			13					
Atef (2015)	240	13	21	0			0		28					
Ellis (2015)	114										48			
Robinson (2015)	28	100	64		7	11	7		64	18				18
Shin (2016)	122	78		25	25		45		3		8			9
Madhu (2017)	25	40	44		8	52	36	20					32	
Rugg (2017)	172	71		19		2	26	4	17	5	12	5		
Nakagawa (2017)	172	65				22					67			
Nakagawa (2019)	72	74									47			28
Eren (2020)	75		92				37		27					
Pooled average	1,920	69	67	13	18	16	23	4	17	8	37	9	14	15
